# Bioactivity comparison of extracts from various parts of common and tartary buckwheats: evaluation of the antioxidant- and angiotensin-converting enzyme inhibitory activities

**DOI:** 10.1186/1752-153X-6-78

**Published:** 2012-08-01

**Authors:** Hweiyan Tsai, Hweiwen Deng, Shangheng Tsai, Yahsien Hsu

**Affiliations:** 1 Department of Applied Chemistry, Chung Shan Medical University, Taichung, 402, Taiwan; 2 Clinical Laboratory, Chung Shan Medical University Hospital, Taichung, 402, Taiwan

**Keywords:** Tartary buckwheat, Common buckwheat, ACE inhibition, Microplate fluometric assay

## Abstract

**Background:**

Buckwheat flour and buckwheat sprouts possess antioxidant properties, and previous studies have reported on buckwheat flour displaying an inhibitory activity for angiotensin-I converting enzyme (ACE). Information is lacking on the bioactivity of other parts of the buckwheat, such as the seed hulls and plant stalks. This study investigates the ACE inhibitory activity and antioxidant activity of various parts of 2 types of buckwheat, namely, common buckwheat (Fagopyrum esculentum Moench) and tartary buckwheat (Fagopyrum tataricum Gaertn).

**Results:**

The extract of common hulls extracted using 50% (v/v)-ethanol solvent presented a remarkable inhibitory activity. The value of IC_50_ is 30 μg ml^-1^. The extracts of both common and tartary hulls extracted using 50% (v/v)-ethanol solvent demonstrated an antioxidant activity that is superior to that of other extracts.

**Conclusion:**

This study determined that the ethanolic extract of the hulls of common buckwheat presented more favorable antioxidant and ACE inhibitory abilities. However, the correlation of antioxidant activity and ACE inhibitory activity for all 18 types of extracts is low. The ACE inhibitory activity could have been caused by a synergistic effect of flavonoids or from other unidentified components in the extracts. The ethanolic extract of common hulls demonstrated remarkable ACE inhibitory activity and is worthy of further animal study.

## Background

Food is not only a source of energy and nutrition for maintenance and growth of the body but is also a source of bioactive compounds that have beneficial effects on humans. For example, common buckwheat (Fagopyrum esculentum) and tartary buckwheat (Fagopyrum tataricum) are traditional foodstuffs available worldwide. They are also essential as functional food resources because of the high polyphenol and mineral contents in the seed. Numerous previous studies have discussed the antioxidant properties of buckwheat flour and buckwheat sprouts [1-12]. Campbell [13] reported that buckwheat comprises 3 classes of flavonoids: flavonols, anthocyanins, and C-glucosyl-flavones. ACE-inhibitory activity of flavonoids isolated from other plants has been reported previously [14,15]. The only reports existent were of dipeptides from buckwheat flour displaying inhibitory activity for the angiotensin-I converting enzyme [16-18]. No information is available regarding the inhibitory activity of angiotensin-I converting enzymes in other parts of the buckwheat plant. From an agricultural perspective, identifying an alternative approach for using the entire buckwheat plant, such as the stalks, leaves, and hulls, is crucial. Therefore, the objective of this study was to evaluate systematically the ACE inhibitory activity and antioxidant activity of different parts and varieties of buckwheat, namely, common buckwheat (Fagopyrum esculentum Moench) and tartary buckwheat (Fagopyrum tataricum Gaertn).

## Results

### The IC_50_ values of various extracts for ACE inhibition

To ensure accurate function of the modified microplate fluormetric assay, we determined the IC_50_ for Captopril, a known ACE inhibitor. Captopril is a common orally administered ACE inhibitor used to treat hypertension and congestive heart failure. The measured IC_50_ was 1.9 ng ml^-1^ (8.7 nM), which was comparable to measurements shown in the literature (7 nM) [19]. Pihlanto-Leppala et al. [19] determined ACE activity by measuring hippuric acid liberated from the reaction of hippuryl-L-histidyl-L-leucine and rabbit ACE with HPLC.

Table 1 shows the IC_50_ of various extracts for ACE inhibition. The rutin equivalent activity was based on the IC_50_ of rutin.

**Table 1:** **The IC**_**50 **_**of varied extracts for ACE inhibition**

**Sample number**	**Portion of buckwheat**	**Extracting solvent**	**IC**_**50 **_**(μg mL**^**-1**^**)**	**Equivalent activity to rutin**
1	Groats, common	Deionized water	283 (±20)	0.30
2	Groats, Tartary	Deionized water	320 (±17)	0.27
3	Groats, common	20%(v/v) ethanol	218 (±11)	0.39
4	Groats, Tartary	20%(v/v) ethanol	237 (±35)	0.36
5	Groats, common	50%(v/v) ethanol	286 (±1)	0.30
6	Groats, Tatary	50%(v/v) ethanol	185 (±5)	0.46
7	Plants, common	Deionized water	629 (±71)	0.14
8	Plants, Tatary	Deionized water	989^a^	0.09
9	Plants, common	20%(v/v) ethanol	646 (±71)	0.13
10	Plants, Tatary	20%(v/v) ethanol	895^a^	0.10
11	Plants, common	50%(v/v) ethanol	331 (±3)	0.26
12	Plants, Tatary	50%(v/v) ethanol	313 (±18)	0.27
13	Hulls, common	Deionized water	424 (±35)	0.20
14	Hulls, Tatary	Deionized water	393 (±40)	0.22
15	Hulls, common	20%(v/v) ethanol	117 (±2)	0.74
16	Hulls, Tatary	20%(v/v) ethanol	168 (±19)	0.51
17	Hulls, common	50%(v/v) ethanol	30 (±2)	2.87
18	Hulls, Tatary	50%(v/v) ethanol	141 (±13)	0.61
control	captopril		0.0019	45263
	quercetin		39	2.21
	rutin		86	1

a: The concentration of IC_50_ is greater than the maximum dissoluble amount. Data were calculated from the linear regression analysis of logarithmic plots.

### The antioxidant activity of various extracts

**Figure 1:**
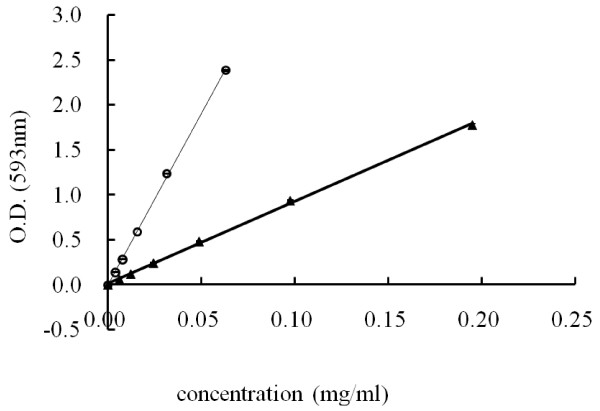
**Dose–response lines for rutin (△) and quercetin (○) solutions. **Three replicates (n = 3) were performed for each concentration.

**Figure 2:**
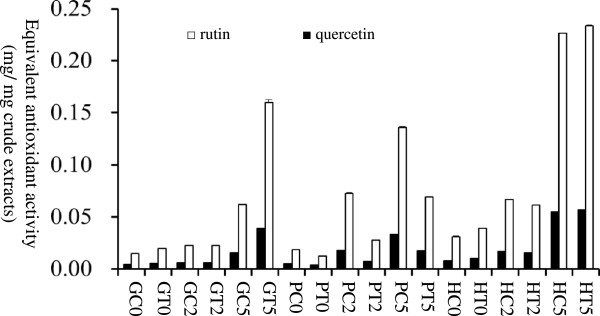
**The equivalent antioxidant activity of various extracts (n = 3).** GC0 is groats of common buckwheat extracted with deionized water. GT0 is groats of tartary buckwheat extracted with deionized water. GC2 is groats of common buckwheat extracted with 20% ethanol. GT2 is groats of tartary buckwheat extracted with 20% ethanol. GC5 is groats of common buckwheat extracted with 50% ethanol. GT5 is groats of tartary buckwheat extracted with 50% ethanol. PC0 is plants of common buckwheat extracted with deionized water. PT0 is plants of tartary buckwheat extracted with deionized water. PC2 is plants of common buckwheat extracted with 20% ethanol. PT2 is plants of tartary buckwheat extracted with 20% ethanol. PC5 is plants of common buckwheat extracted with 50% ethanol. PT5 is plants of tartary buckwheat extracted with 50% ethanol. HC0 is hulls of common buckwheat extracted with deionized water. HT0 is hulls of tartary buckwheat extracted with deionized water. HC2 is hulls of common buckwheat extracted with 20% ethanol. HT2 is hulls of tartary buckwheat extracted with 20% ethanol. HC5 is hulls of common buckwheat extracted with 50% ethanol. HT5 is hulls of tartary buckwheat extracted with 50% ethanol.

Figure 1 illustrates the reductive capabilities of rutin and quercetin. These calibration curves were used to calculate equivalent activity of various extracts related to rutin or quercetin (Figure 2). The equivalent activities of rutin and quercetin are perfect positive correlation (correlation r = 1) for 18 samples. Performing the FRAP assay is quick and simple, and the reaction is reproducible and linearly related to the weight concentration of the flavonoids. The crude extracts of tartary and common hulls extracted with 50% ethanol presented the greatest antioxidant activity. The activity per milligram of crude extract was equivalent to 0.23 mg of rutin or 0.055 mg of quercetin.

## Discussion

By comparing the extracts of the various extracting solvents, we discovered that extracts obtained from 50% v/v ethanol solvent exhibited the greatest ACE inhibitory activity. We evaluated the ACE activity of various extracts using a paired *t* test. The results showed that various ethanolic extracts presented significant differences of ACE inhibitory activities for all parts of the plant, except for the groats from common buckwheat, where the choice of solvent made no significant difference. Extracts from buckwheat plants (comprising stalks, stems, and leaves) showed poor ACE inhibitory activity for all solvents.

For the extracts with deionized water, groats presented optimal ACE inhibitory activity (lowest IC_50_ values), as compared to the plants and hulls. The ACE inhibitory activity of water-extracts could be caused by water-soluble peptides. This was consistent with the findings reported by Aoyagi et al. [16]. The buckwheat flour contained water-soluble peptides, which inhibited ACE activity. The 50% ethanolic extract of common hulls produced the greatest inhibitory activity. The value of IC_50_ (30 μg ml^-1^) was significantly lower than the IC_50_ values of other extracts. Li et al. [17] reported that proteins of tartary buckwheat flour treated with pepsin, followed by chymotrypsin and trypsin hydrolysis, resulted in an ACE inhibitory activity with IC_50_ at 140 μg ml^-1^. Therefore, ethanolic-soluble ingredients demonstrate an inhibitory activity that is superior to that of water-soluble ingredients.

**Figure 3:**
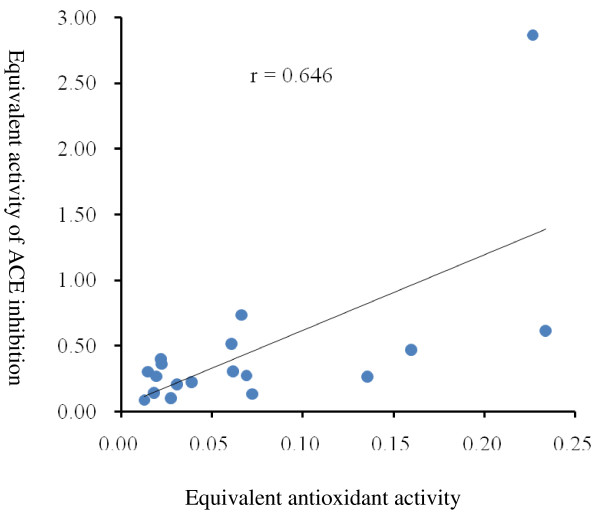
**Correlation plots of equivalent antioxidant activity and ACE inhibitory activity of 18 samples. **The equivalent activities were based on the activity of pure rutin.

Figure 3 shows the correlation plots of equivalent antioxidant activity and ACE inhibition. Comparing the rutin equivalent antioxidant activity and ACE inhibitory activity using the Pearson correlation showed that the correlation coefficient was 0.6, indicating that the correlation between these 2 bioactivities was low to medium. Quercetin and rutin, which are 2 major types of flavonoids in buckwheat [20], presented great antioxidant activity. We evaluated the inhibitory effect of commercially available quercetin and rutin. The IC_50_ values were 39 μg ml^-1^ and 86 μg ml^-1^ for quercetin and rutin, respectively. The antioxidant activity of crude extracts of common hulls per milligram was equivalent to 0.055 mg of quercetin only; thus, the high ACE inhibitory activity of the crude extracts could be caused by other unidentified components in the extracts or by a synergistic effect of flavonoids, as suggested by Lacaille-Dubois et. al. [14].

However, the presence of strong in vitro activities does not necessarily imply that hull extracts can provide powerful anti-hypertensive and antioxidant drugs. These preliminary bioactive results of ethanolic extracts of common hulls warrant additional animal studies.

## Materials and methods

### Materials

Captopril was obtained from ICN biomedical Inc. (Costa Mesa, CA, USA). Angiotensin-converting enzymes from porcine kidneys (EC 3.4.15.1), rutin hydrate, isoorientin, and quercetin anhydrous were purchased from Sigma (St. Louis, MO, USA). O-phthaldialdehyde reagent (OPA) containing OPA (1 mg ml^-1^) with 2-mercaptoethanol as a sulfhydryl moiety was obtained from Sigma. All chemicals were used without further purification.

Common buckwheat (Fagopyrum esculentum) and tartary buckwheat (Fagopyrum tataricum) were sown in autumn in Erlin Township, Changhua County, Taiwan. The grains were harvested approximately 100 d after sowing.

A microplate fluometric assay was measured using a Flex Station 3-microplate reader (Molecular Devices, Sunnyvale, CA, USA).

#### Preparation of the extracts

According to the regulations of the Department of Health, Taiwan, solvents extracted for functional foods or medicinal herbs may contain up to 50% (v/v) ethanol. Thus, we extracted various buckwheat plant samples using 3 different solutions: deionized water; 20% (v/v) ethanol; and 50% (v/v) ethanol, at 60°C for 2 h.

The grains were dehulled manually and divided into groats and hulls. The buckwheat plants (comprising leaves, stems, and stalks), groats, and hulls were ground separately. Thirty-gram aliquots of each ground sample were extracted using 300 ml of the various extracting solvents, under constant magnetic stirring in a 60°C water bath for 2 h. After extraction, the slurry was filtered, and subsequently centrifuged at 5000 rpm for 20 min. The supernatant was collected and frozen at −20°C and concentrated to dryness under reduced pressure. The dried crude extracts were stored at −20°C. Fresh solutions were prepared before each assay. The crude extracts were reconstituted using the respective extracting solvents, and then diluted to various concentrations using an ACE assay buffer.

### Angiotensin-I-converting enzyme inhibitory assay

This study used a microplate fluometric assay modified according to the method proposed by Schwager et al. [21] to provide rapid screening and to consume fewer reagents. The fluorometric assay involved using the hydrolysis of hippuryl-L-histidyl-L-leucine (HHL) by ACE to form hippuric acid and histidyl-leucine (HL). Adding aqueous NaOH stopped the reaction. The product HL was reacted with OPA [22], and fluorescence was detected at an excitation of 365 nm and an emission of 460 nm.

A lyophilized angiotensin-converting enzyme derived from porcine kidneys was reconstituted with an assay buffer to a concentration of 12.5 mU ml^-1^. The assay buffer (pH 8.3) comprised potassium di-hydrogen phosphate (0. 1 M), di-sodium hydrogen phosphate (0.1 M), and zinc chloride (10.0 mM). A brief description of the assay is as follows: the ACE solution (20 μl) was incubated using an assay buffer (50 μl) containing HHL (5.0 mM) and various concentrations of buckwheat extracts (10 μl). The reaction was conducted at 37°C for 30 min, and then stopped by adding aqueous NaOH (50 μl, 1.0 M). An OPA reagent (50 μl) was reacted with the HL produced from enzymatic hydrolysis for 10 min, and fluorescence intensity was subsequently measured at 365 nm excitation and 460 nm emission. To correct for intrinsic fluorescence of ACE and the extracts, a blank was prepared by adding NaOH immediately after adding ACE, followed by adding HHL (50 μl) and the extract solution (10 μl).

The relative ACE activity was calculated as follows:

$$\begin{array}{l} \text{relative ACE activity}\%\\ =\frac{\text{Flourescence intensity of inhibitory experiments - blank}}{\text{Flourescence intensity of 100\% activity assay - blank}} \end{array}$$

The relative ACE activity% versus the logarithmic concentrations was plotted. The concentration of 50% inhibition of ACE activity (IC_50_) was determined for varied extracts by using linear regression analysis of logarithmic plots. We conducted at least 3 replicates for each extract.

### Ferric-reducing antioxidant power assay

The antioxidant activities of the extracts were determined according to the procedures recommended by Benzie and Strain [23]. In brief, 40 μl of flavonoid-containing solutions were mixed in a 96-well plate with 300 μl of a reagent solution containing 0.8 mM of tripyridyltriazine and 1.7 mM of FeCl_3_ in 300 mM sodium acetate (pH 3.6). The samples were incubated for 15 min at 37°C, and the absorbance at 593 nm was recorded on a Flex Station 3-microplate reader.

### Statistical analysis

Statistical differences between the groups were evaluated using the *t* test for paired data. The *p*-values were considered significant at *p* < 0.05. We used Microsoft Excel® 2007 software for statistical analysis.

## Conclusion

In conclusion, the findings showed that the ethanolic extract of hulls of common buckwheat presented powerful anti-hypertensive and antioxidant properties. It is worthy of further animal study and further investigation on components of the extract.

## Authors’ contributions

HY made substantial contributions to the conception and design, analysis and interpretation of data, and drafting and revision of the manuscript. HD performed the design and data collection of ACE inhibitory experiments. ST conducted the sample extraction. YH performed the FRAP assay. All authors read and approved the final manuscript.
